# National Study on Mental Health and Emotional Wellbeing among Young People in Malta: Phase 1

**DOI:** 10.1192/j.eurpsy.2022.1527

**Published:** 2022-09-01

**Authors:** R. Sacco, N. Camilleri, D. Newbury Birch

**Affiliations:** 1 Malta Mental Health Services, Cyps, Attard, Malta; 2 Teesside University, Social Sciences, Teesside, United Kingdom

**Keywords:** Epidemiology, Prevalence, young people, Malta

## Abstract

**Introduction:**

Half of all mental disorders (MD) begin by age 14, however, the majority of disorders remain untreated well into adulthood due to inadequate service provision. Prevalence studies of MD among young people (YP) are needed to elucidate the current epidemiology and better service development to prevent and help YP with MD in the Maltese islands. This abstract describes the first phase of a 3-phase national study.

**Objectives:**

1. To screen for MD among a sample of 5–16-year-olds. 2. To determine the presence or absence of a range of protective and risk factors among YP with and without a MD.

**Methods:**

A multi-stage random sample of 800 YP aged 5-16 years were recruited from 39 schools across the Maltese Islands. Participants were screened for MD using the SDQ, SCARED, AQ10, SCOFF and AUDIT, and asked questions on life experiences.

**Results:**

25.2% of YP were identified as being at risk of suffering from a MD (T1). Only 10% of these were referred to MHS. A greater proportion of YP identified as having a possible MD (compared to those without), were found to have a physical impairment (19%), problematic family dynamics (12%), adverse life events (T2) and parents with a history of health/social problems (T3).

**Figure:**
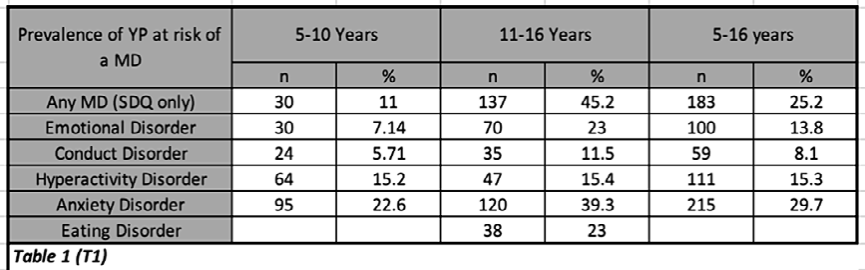

**Figure:**
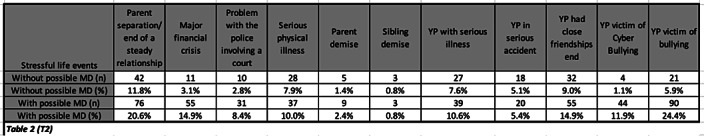

**Figure:**
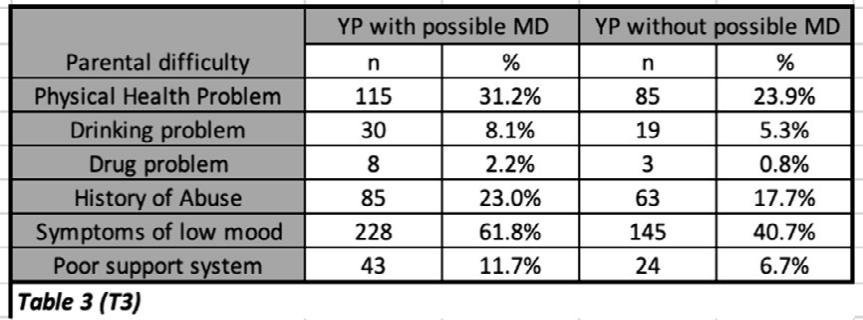

**Conclusions:**

The K-SADS will be conducted on YP identified as having a possible MD to ascertain a categorical diagnosis and establish prevalence rates for MDs as defined by DSM-5 criteria. Recommendations to improve and develop new mental health services to meet the needs for these YP will be disseminated amongst commissioners.

**Disclosure:**

No significant relationships.

